# Application of Milk Exosomes for Musculoskeletal Health: Talking Points in Recent Outcomes

**DOI:** 10.3390/nu15214645

**Published:** 2023-11-01

**Authors:** Na-Hyung Kim, Juhae Kim, Joo-Yeon Lee, Hyeon-A Bae, Choon Young Kim

**Affiliations:** 1 Research Institute of Human Ecology, Yeungnam University, Gyeongsan 38541, Gyeongbuk, Republic of Korea; knahyu@yu.ac.kr (N.-H.K.); kimjh825@yu.ac.kr (J.K.); jooyeonlee@ynu.ac.kr (J.-Y.L.); bha0720@yu.ac.kr (H.-A.B.); 2 Department of Food and Nutrition, Yeungnam University, Gyeongsan 38541, Gyeongbuk, Republic of Korea

**Keywords:** milk exosomes, human milk, bovine milk, camel milk, canine milk, musculoskeletal disorders, health, bone, muscle, sarcopenia

## Abstract

Milk is a nutrient-rich food source, and among the various milks, breast milk is a nutrient source provided by mothers to newborns in many mammals. Exosomes are nano-sized membranous extracellular vesicles that play important roles in cell-to-cell communication. Exosomes originate from endogenous synthesis and dietary sources such as milk. Discovered through electron microscopy as floating vesicles, the existence of exosomes in human milk was confirmed owing to a density between 1.10 and 1.18 g/mL in a sucrose gradient corresponding to the known density of exosomes and detection of MHC classes I and II, CD63, CD81, and CD86 on the vesicles. To date, milk exosomes have been used for treating many diseases, including cancers, and are widely proposed as promising carriers for the delivery of chemotherapeutic agents. However, few studies on milk exosomes focus on geriatric health, especially sarcopenia and osteoporosis related to bone and muscle. Therefore, the present study focused on milk exosomes and their cargoes, which are potential candidates for dietary supplements, and when combined with drugs, they can be effective in treating musculoskeletal diseases. In this review, we introduce the basic concepts, including the definition, various sources, and cargoes of milk exosomes, and exosome isolation and characterization methods. Additionally, we review recent literature on the musculoskeletal system and milk exosomes. Since inflammation and oxidative stress underly musculoskeletal disorders, studies reporting the antioxidant and anti-inflammatory properties of milk exosomes are also summarized. Finally, the therapeutic potential of milk exosomes in targeting muscle and bone health is proposed.

## 1. Introduction

Exosomes are nano-sized extracellular vesicles (EVs), which are small membranous vesicles originating from various cells and tissue [1,2]. Exosomes, secreted from various cells, are released into bodily fluids after the fusion of multivesicular bodies and the plasma membrane. Exosomes are a type of EV, along with microvesicles, originating from the plasma membranes and apoptotic bodies derived from apoptotic cells. Among EVs, exosomes are the smallest, measuring approximately 30–150 nm [3]. As a cargo of proteins, lipids, and nucleic acids, exosomes contain many constituents of a cell, with the capability of delivering these cargoes to recipient cells; they may also reprogram the targeted cells from their release [2,4].

To date, exosomes have been isolated in many kinds of body fluids including blood, urine, saliva, breast milk, amniotic fluid, ascites, cerebrospinal fluids, and semen [5,6]. Among them, breast milk, which can also be defined as food, has received great attention for the wide range of volumes in which it is produced by various mammals [7]. Milk exosomes have been used as drug carriers for the treatment of many diseases [8]. Most of these are anti-cancer drugs, and milk-derived exosomes have been widely proposed as promising carriers for the delivery of chemotherapeutic agents [9] including celastrol, a representative case of lung cancer [10]. Natural compounds such as curcumin [11] and berry anthocyanins [12] have also been tested in ovarian and colorectal cancer models. A recent review suggested that milk exosomes mainly act against hypoxia, immune responses, intestinal diseases, and cancer. Milk-derived exosomes exhibit good stability [13], low immunogenicity, and excellent cross-specificity tolerance [14]. In addition, milk itself has various health-beneficial constituents such as β-lactoglobulin, α-lactalbumin [15], milk fat globule membrane [16], and milk oligosaccharides [17]. Thus, in addition to being potentially excellent drug carriers, milk exosomes also play a positive role in human health. For instance, milk exosomes subfractioned from human milk are abundant in small interfering RNA (siRNA)-148a, which plays an important role in immune function and epigenetic regulation [18].

**Figure 1:**
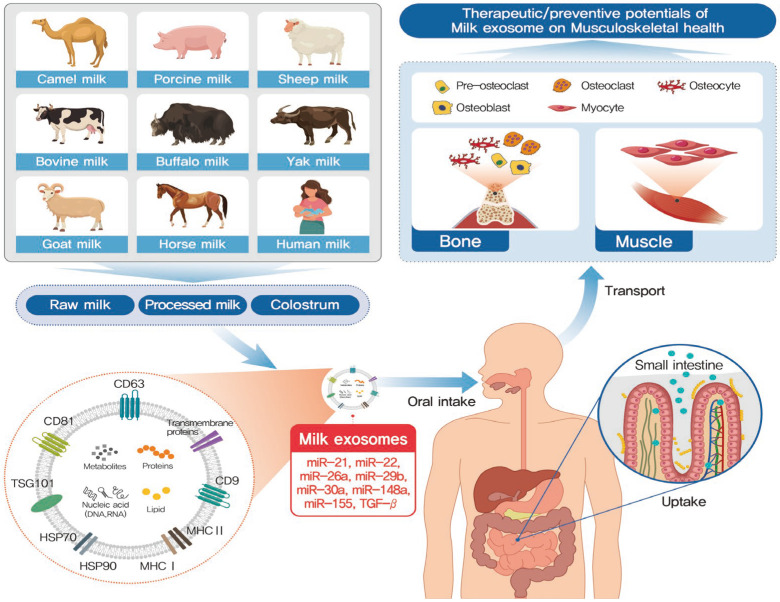
Summary of milk exosomes on musculoskeletal health.

**Figure 2:**
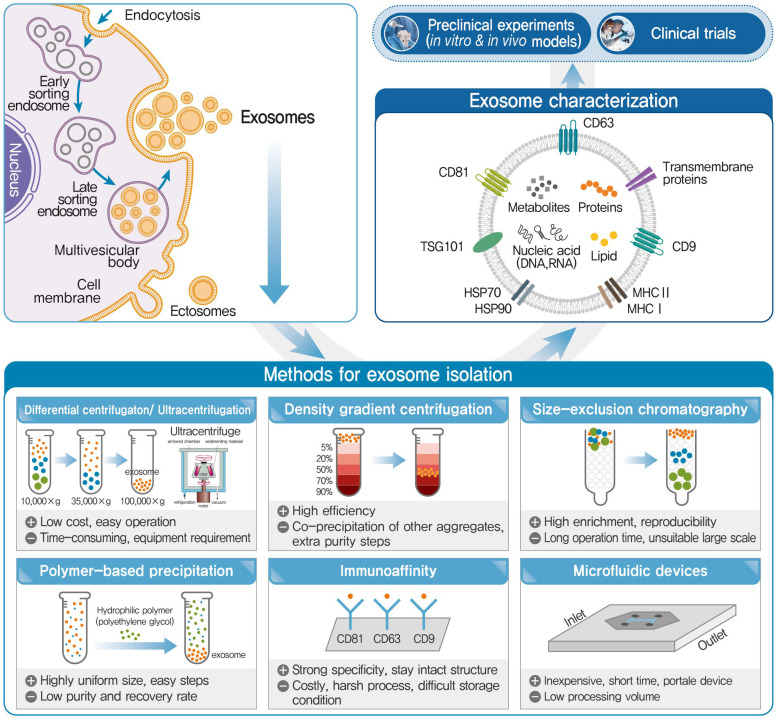
Isolation and characterization of milk exosomes.

Few studies have been conducted using milk exosomes with a focus on geriatric health, especially sarcopenia and osteoporosis. Although a recent review discussed the role of bioactive molecules in extracellular vesicles in the pathophysiological processes of skeletal muscle atrophy [19], studies focusing on milk-derived exosomes are scarce. As of October this year, about 20 recent studies on the musculoskeletal system of exosomes have been published rapidly (accessed on 23 October 2023 to National Center for Biotechnology Information), and many research results on milk exosomes that can be safely consumed are expected to be published soon. Therefore, this review aims to provide evidence for the potential of milk exosomes in preventing and/or managing sarcopenia and osteoporosis (Figure 1). First, the basic concepts of milk exosomes, including their sources, cargoes, and isolation and characterization methods, are explained (Figure 2). Previous reports targeting muscle and bone health using milk exosomes are also reviewed. Because inflammation and oxidative stress are the underlying cause of musculoskeletal disorders, studies reporting the antioxidant and anti-inflammatory properties of milk exosomes are summarized. Finally, the therapeutic potential of milk exosomes in targeting muscle and bone health is proposed.

## 2. Milk Exosomes

### 2.1. Definition of Milk Exosomes

Milk is a nutrient-rich food source provided by mothers to newborns in many mammals. Exosomes in breast milk were detected through electron microscopy as floating vesicles with a density between 1.10 and 1.18 g/mL (in a sucrose gradient), which corresponded to the known density of exosomes. Moreover, MHC classes I and II, CD63, CD81, and CD86 were detected on the vesicles, confirming experimentally that exosomes exist in human milk [20]. Milk membrane glycoproteins and milk fat globule-epidermal growth factor (MFG-E) 8 are abundantly expressed in lactating mammary glands as a component of the exosome [21].

### 2.2. Source of Milk Exosomes

Milk exosomes are derived from various sources, including the human breast, cow, buffalo, camel, goat, sheep, yak, and porcine [6]. Among these sources of milk exosomes, it is reported that exosomes derived from human breast milk influence immune responses since breast milk contains a variety of components including milk fat globules, immune competent cells, immunoglobulin A, cytokines, and antimicrobial peptides [20]. HLA-DR and the tetraspanin molecules (CD63, CD81) were expressed on surface proteins of exosomes from colostrum and mature milk [20]. Comparison studies of process-based milk exosomes have also been conducted. Profiles of miRNA at different stages of human lactation (colostrum, transitional, and mature milk) revealed the dynamic changes in miRNA composition over time [22,23]. One study revealed that the number of exosomes is the highest in colostrum compared to the milk produced at other stages [23]. In addition, storage at −80 °C did not affect the exosome-sized vesicle count in human milk for 4 weeks [24]. Similarly, milk frozen at −80 °C can be isolated by producing exosomes and their miRNA cargo [25]. Furthermore, milk pasteurization decreases its immunomodulatory activity and integrity [26]. Numerous studies have been conducted on milk exosomes derived from cows. The production of bovine milk exosomes is scalable and cost-effective; 1 mL of milk yields 10^12^ BME-sized extracellular vesicles [27]. Several studies have focused on the cargo ingredients from milk exosomes. Analysis of the bovine milk protein database revealed that among different milk fractions, including whey, fat globule membranes, and exosomes, exosomes contained 684 exclusive proteins from an entire list of 6063 proteins [28]. Proteins can be post-translationally modified by glycosylation; some glycosylated proteins are associated with diseases and can serve as potential biomarkers. Compared to whey, analysis of bovine milk exosomes revealed that 114 glycoproteins were located on their surface, and two sialylated and lacdiNAc N-glycans were found to be potential biomarkers for bovine milk exosomes [29]. Genome-wide miRNA profiling analysis was performed on milk exosomes from healthy cows (3–4-year-old Chinese female Holstein cattle). A total of 1472 miRNAs were identified via high-throughput sequencing; among them, 492 were known bovine miRNAs and 980 were novel miRNAs. Further, ten highly expressed miRNAs accounted for 80–90% of the total reads, and there were no scRNAs or few snRNAs in bovine milk exosomes [30]. In addition, bovine milk contains immune-related miRNAs, such as miR-15b, miR-27b, miR-34a, and miR-10. These miRNAs are particularly abundant in colostrum [31]. Bovine-specific miR-2478 has been reported as the most abundant miRNA in milk exosomes [32]. Some studies have focused on the process of milk exosome production. During ultrasonication, 76% of the exosomes are lost, and the uptake of the remaining exosomes is 40% less efficient than that of non-ultrasonicated exosomes [33]. Ultra-heat treatment of milk diminishes milk exosomes, resulting in the absence of intact EVs. Pasteurization also affects milk EVs, with a loss of milk EV-associated RNAs without affecting the number of EVs [34]. Commercial processing, including heat treatment and pasteurization, affects ncRNA expression, but the overall abundance of all miRNAs was not significantly different from that in raw (untreated) milk exosomes [35]. Comparison between milk at different stages showed that colostrum-derived exosome contains miRNA, which are mostly involved in immunity pathways [36].

In addition to human- and cow-derived exosomes, milk exosomes from other species including buffalo, camel, goat, and porcine have been investigated. Milk from water buffaloes (Murrah breed) contributes about 13% to the milk production in the world and contains rich nutrients including fat, unsaturated fatty acids, proteins, amino acids, vitamins, mineral, and membrane-bound carriers such as exosomes, which were recently compared with cow milk [37]. The most abundant of miRNAs was bta-Mir-148a, accounting for about 38.9% in each buffalo library, consistent with cow, porcine, and human milk. Rani et al. reported that Bta-miR-148a, bta-miR-30a-5p, bta-miR-21-5p, bta-miR-99a-5p, bta-miR-27b, bta-miR-200a, bta-miR-26a, bta-miR-26c, bta-let-7g, and bta-let-7i were the 10 most abundant microRNAs in buffalo milk exosomes [38]. Similarly, buffalo-milk-derived exosomes expressed miR-21 and miR-500 that were found stable under different household storage conditions, together with miR-15b, miR-27b, miR-125b, and miR-155 [39].

The daily drinking of camel milk in the Middle East is known to maintain health by improving immune function since kappa casein and lactoferrin are included in camel milk [40]. The CD63 and CD81 are expressed in exosomes derived from camel milk at four lactation stages: colostrum (from birth to 3 days postpartum), early (from 10 to 99 days), mid (from 100 to 200 days), and late (after 200 days postpartum) lactation [40]. In addition, a previous report demonstrated that camel milk exosome treatment elevated CD4^+^ and CD8^+^ T cells in the spleen of MCF7-bearing rats, and represented inhibition of inflammation, immunosuppression, or oxidative stress, and enhancement of tumor cell apoptosis [41].

Goat-milk-derived exosome is notable as a cost-effective source of exosomes and was reported to exert significant effects on the inflammatory pathway [42]. However, the limitation in using goat-milk-derived exosomes, from a biomedical perspective, is that they have a relatively high fat content compared to exosomes derived from various other milk sources consumed by humans [42,43]. Santos-Coquillat et al. suggested the promising use of goat milk exosomes as natural probes in the detection of inflammatory processes, after goat milk exosomes are isolated and fully characterized [43].

A previous study demonstrated that the porcine colostrum is mainly composed of protein, carbohydrates, lipids, lesser minerals, vitamins, leukocytes, somatic cells, bacteria, and exosomes [44]. Additionally, porcine colostrum exosomes are enriched with immune-related microRNA, whose concentration in serum was higher in newborn piglets fed colostrum than in those fed mature milk [44]. The bioactive effects of milk exosomes from various sources were investigated and summarized in more detail in the 2.4. Biological activities of milk exosomes.

### 2.3. Cargo of Milk Exosomes

The cargo of milk exosomes refers to the molecules contained inside the exosomes, which are small endoplasmic reticulum molecules that are formed inside cells and secreted [45]. Milk exosomes primarily comprise extracellular molecules. These molecules may be related to specific functions in cells that secrete exosomes and may also perform various physiological functions in recipient cells [45,46,47]. The cargo of milk exosomes may include molecules such as RNA, proteins, and lipids, and milk exosomes. They may include a wide variety of proteins that perform functions such as cell signaling, cell adhesion, and regulation of immune responses [45,48]. The membranes of milk exosomes may vary depending on the type of exosome-producing cells, individual differences, and physiological conditions. Thus, the study of the cargo in milk exosomes plays an important role in understanding the properties of the cargo molecules and biological functions of exosomes. These milk exosome cargoes perform various functions and biological actions. Several major cargo molecules, including those that transport growth factors and signaling proteins, contain anti-inflammatory and immunomodulatory proteins and nucleic acid and lipid molecules [45].

Transported growth factors and signaling proteins can reach recipient cells and regulate physiological processes such as cell division, cell survival, and tissue regeneration [48]. Growth factors affect cell survival, growth, division, and differentiation. Milk exosomes carry growth factors and deliver them to the recipient cells [48]. Growth factors bind to cell surface receptors and initiate signal transduction, which activates intracellular signaling pathways and induces physiological changes in the cells [48]. Growth factors contribute to various physiological functions, including cell growth, tissue regeneration, immune response regulation, and angiogenesis [48]. Signaling proteins are involved in both intracellular and intercellular signal transduction [47,48]. Milk exosomes carry a wide variety of signaling proteins that are delivered to recipient cells to activate or inhibit specific signaling pathways [46]. These signaling proteins regulate the physiology, growth, differentiation, and survival of cells, and can often influence inflammatory regulation, immune responses, and cell motility [48].

The cargo-related anti-inflammatory and immune modulating proteins contribute to the regulation of the immune system by performing roles such as regulating the immune response, suppressing inflammation, and presenting immune antigens [45,49]. Milk exosomes contain inflammatory regulatory proteins. Inflammation is part of the immune response and can be caused by tissue damage, infection, or autoimmune responses [45,49]. Inflammation regulatory proteins play an important role in the inflammatory process and can be delivered to receptor cells via exosomes to suppress or modulate inflammatory responses. These proteins play a role in suppressing or regulating inflammation through the production of inflammatory mediators, regulation of inflammatory signaling pathways, and regulation of inflammatory responses [45,49]. Milk exosomes may contain immunomodulatory proteins. These proteins balance the immune system and prevent the autoimmune responses from becoming overactive. Immunomodulatory proteins transported through milk exosomes contribute to immune regulation by reducing or inducing the appropriate responses.

Nucleic acid cargoes, such as miRNAs, are important cargoes of exosomes that affect cell function regulation by participating in transcriptional regulation in recipient cells [50,51]. Nucleic acids play an important role in milk exosome cargo. These molecules reside inside exosomes and are transported to recipient cells. Nucleic acids are present within exosomes, mainly in the form of RNA. RNA carries information from DNA and plays important roles in regulating gene expression and cellular functions [50,51]. RNAs, such as mRNAs, miRNAs, and lncRNAs, are translated or regulated within the recipient cell and may affect the gene expression and regulation of cell function. Transfer of gene information from DNA to proteins occurs through mRNA, and the mRNA contained in milk exosomes is translated into recipient cells, influencing protein synthesis. Small RNAs, such as miRNA, are involved in the regulation of gene expression and are delivered to recipient cells as cargo to inhibit or regulate the expression of specific genes. As exosomal cargoes, miRNAs regulate the expression of more than 60% of human genes [52].

Unlike mRNA, lncRNA is a non-messenger RNA that does not play a direct role in protein synthesis, but may be involved in regulating various cell functions and gene expression, and is known to be included in milk exosomes [46]. When nucleic acids contained in milk exosomes are delivered to recipient cells, they perform specific functions in the recipient cells. Protein synthesis is regulated by mRNA, whereas miRNAs and lncRNAs regulate gene expression and cell function [50,51].

Milk exosomes embed lipid molecules in their membrane structures [53]. Lipids not only play an important role in maintaining exosome stability and structure but also contribute to the transport of cargo molecules and their delivery within the recipient cell [54]. These lipids contribute to the protection and transport of cargo molecules while maintaining the stability and structure of exosomes [54]. Lipid components also play important roles in the binding and internal delivery of exosomes to recipient cells. Phospholipids distinguish the inside and outside of exosomes and play a role in stably embedding and transporting cargo molecules, whereas cholesterol maintains the stability of exosomes and serves as a structural support [53,54]. Cholesterol also plays a role in regulating interactions between exosomes and receptor cells [53,54]. Lipid-lytic enzymes metabolize and degrade lipid molecules in exosomes to make the transported cargo molecules available to recipient cells [47].

### 2.4. Biological Activities of Milk Exosomes

As a variety of studies have already reported, this subsection is summarized on the beneficial health effects of milk exosomes from major sources described previously. Several studies have been conducted on exosomes derived from human breast milk. First, the beneficial effects of human-breast-milk-derived exosomes on intestinal health were reported. Milk exosomes protect intestinal epithelial cells against oxidative stress [55]. They also decrease inflammation in hypoxic and lipopolysaccharide-treated intestine organoids [56]. Necrotizing enterocolitis (NEC) is a serious gastrointestinal disease mostly affecting premature babies, which is ameliorated with breastfeeding [57]. A human-milk exosome study reported that milk exosomes exert beneficial effects by reducing inflammation and injury to the intestinal epithelium, as well as by restoring intestinal tight-junction proteins [58,59]. Among the exosomes obtained from colostrum, transitional, and mature milks, colostrum-milk-derived exosome showed the strongest protective effects against intestinal injury [60]. Furthermore, exosomal circular RNAs (circRNAs) were found to be a key player in promoting vascular endothelial growth factor protein expression, inducing the proliferation and migration of small intestinal epithelial cells [61]. In addition, milk exosomal miR-148a-3p and its upregulation of p53 and sirtuin1 have been suggested as key factors and pathways involved in the protection against NEC [62]. In in-depth analyses including lncRNA and mRNA profiling, peptidomics, and lipidomics, milk exosomes play critical roles in protecting against NEC [63,64,65]. The analysis was conducted by comparing breast milk exosomes from healthy lactating mothers who delivered term and preterm infants. Preterm milk samples showed significantly different profiles, indicating their potential regulatory roles in intestinal epithelial cell function. A comparison between preterm and term milk exosomes was also conducted. Preterm milk exosomes can be preserved in the gut following simulated gastric/pancreatitis digestion. Although the abundance of microRNA (miRNA) was lower in preterm milk exosomes than in term milk, the composition of term and preterm milk was comparable [66]. The effect of mother’s condition on milk exosome composition was also tested. The content of individual miRNAs and other non-coding RNA in milk exosomes depends on the mother’s diet [67]. Milk exosomes from mothers with type 1 diabetes have also been investigated, showing aberrant levels of miRNAs [68,69,70] as well as exhibiting distinct regulatory bioactivities on hepatocyte proliferation, with the inhibition of hepatocyte proliferation by downregulating mTOR via exosomal miR-101-3p delivery [71]. Milk exosomes from obese mothers also have altered miRNA composition, whose target genes are associated with neurological diseases and psychological disorders, indicating an impact on breastfed infants [72]. Specifically, miRNA-148a and miRNA-30b from exosomes in milk from obese mothers have been revealed to be significant predictors of infant growth and fat acquisition in early infants, by association analysis [73]. In addition to its protective effects on intestinal health, milk exosomes protect against infectious agents [74] and neonatal bronchopulmonary dysplasia [75] and contributes to optimal neuronal development and brain health [76]. The effect of milk exosomes has been reported with specific/identified target cargo from exosomes. Kim et al. [77] reported that bovine-colostrum-derived exosomes promote hair regeneration by accelerating the hair cycle transition from the telogen to anagen phase. Lactoferrin, which is highly expressed in milk exosomes, as shown by western blot analysis, has been suggested as a mediator of this effect, as lactoferrin is also known as a hair growth promoter and anti-aging agent. The authors also suggest that not only lactoferrin but also other bioactive compounds such as various miRNAs can mediate the Wnt/β-catechin pathway, which has been shown to affect hair loss [77]. Bovine-specific miR-2478 in milk exosomes has been shown to suppress melanogenesis through the Akt-GSK3beta pathway [78], implying that milk exosomes can be a useful cosmeceutical ingredient to improve skin whitening. The effect of dietary bovine milk exosomes on bacterial communities in mice has also been reported [79]. In addition, several studies have demonstrated the therapeutic effects of bovine milk exosomes in various diseases. In the intestine, the preventive effect of milk-derived exosomes against the development of necrotizing enterocolitis, which is characterized by intestinal injury and impaired mucin synthesis, is mediated by the promotion of goblet cell expression/production and ER functioning [80]. Attenuation of inflammasomes in the lungs during neonatal NEC has also been demonstrated by treatment with bovine milk exosomes [81]. The alleviation of cardiac fibrosis and enhancement of cardiac function in rats with cardiac fibrosis by enhanced angiogenesis have also been reported [82]. The potential effect of milk exosomes on the immune system has also been suggested by the enhanced proliferation of RAW264.7 macrophages and protection against cisplatin-induced cytotoxicity following exosome treatment [83]. Similarly, enhancement of macrophage proliferation under hypoxia by milk exosomes has been reported [84]. Effects on scar-free wound healing by milk exosomes have also been reported, based on TGF-β1 signal regulation [85]. Useful effects on anti-cancer, antioxidant, and anti-renal-injury-related effects of camel-milk-derived exosomes were reported in five studies. While four studies utilized milk from the mid-lactation period, one compared the effectiveness depending on the lactation period, revealing that colostrum had the highest anti-cancer effect in a human liver cancer model [40]. In addition, only one study has shown a major contribution to exosome function, including that of lactoferrin and kappa casein, the protein cargo [41]. Some studies have used buffalo-derived milk exosomes. Two of the studies were conducted using sequencing methods. Chen et al. reported that the miRNA profiles from exosomes obtained from buffalo milk during the mid-lactation period were enriched in the modulation of disease resistance, immune responsiveness, and basic metabolic events [37]. Performing in silico analysis, they revealed that ubiquitin proteasomal degradation is the process most regulated by buffalo milk exosomal miRNAs [38]. A previous study reported an immune miRNA signature in exosomes isolated from buffalo milk. This study also revealed that the immune miRNA concentrations in exosomes were higher than those in other body fluids, serum, and urine [39]. In a recent study, in addition to profiling the exosome-derived miRNAs from buffalo milk, miR-27b has been suggested as being a supportive anti-cancer effect contributor, by demonstrating aggravation of apoptosis rate, mitochondrial stress, and endoplasmic reticulum in colorectal cancer cell models and both HCT116 and HT-29 cell lines, under conditions of miR-27b overexpression [86].

## 3. Isolation and Characterization of Milk Exosomes

### 3.1. Isolation Methods

#### 3.1.1. Differential Centrifugation and Ultracentrifugation

Differential centrifugation and ultracentrifugation are the most commonly used methods for exosome isolation and require multiple experimental steps [87,88]. This method is based on a series of different centrifugal forces and times, which result in the sedimentation of exosomes, proteins, cells, and other substances in the sample [89]. First, a low-speed spin (10,000× *g*) allows the isolation of apoptotic bodies and larger vesicles. Then, a higher speed (approximately 35,000× *g*) is used to remove the macrovesicles. Finally, exosomes are precipitated by ultracentrifugation (approximately 100,000× *g*) [89]. Differential centrifugation is easy, convenient, and inexpensive [88,90]. However, the method is not suitable for isolating nanovesicles from milk because of the very low purity achieved [88,89]. Based on experiments comparing the number of milk exosome particles collected, Morozumi et al. reported that ultracentrifugation resulted in a significantly lower number of milk exosomes than that by other isolation methods [91]. In addition, ultracentrifugation is time-consuming and can damage the membranes and shape of exosomes [6,88].

#### 3.1.2. Density Gradient Centrifugation

Density gradient centrifugation uses a gradient media for exosome separation [6,89]. The principle of this method is similar to that of differential centrifugation except those crude exosomes must be placed in a density gradient medium and centrifuged for further purification [89]. Furthermore, this method is more effective than the conventional differential centrifugation [89]. Density gradient centrifugation achieves a much greater separation efficiency than the conventional method, resulting in a higher yield of isolated exosomes [6,89]. Therefore, this method could be used to isolate exosomes with protective physicochemical properties [89]. Yamada et al. reported that density gradient centrifugation with ultracentrifugation yielded milk exosomes with intact native morphology [92]. However, this method is limited by the coprecipitation of protein aggregates, apoptotic bodies, and nucleosomal fragments during isolation, leading to low exosome purity [6].

#### 3.1.3. Size-Exclusion Chromatography

Size-exclusion chromatography (SEC) is a chromatographic method in which EVs of different diameters are separated using a gel column [93]. The SEC principle is based on the relationship between the pore size of the stationary phase and the particle size [93]. Extracellular vesicles with larger sizes elute first and travel quicker than the smaller ones, which can precipitate together with protein aggregates [88]. The advantages of SEC over differential centrifugation include simplicity, higher enrichment, better reproducibility, and higher purity [93]. However, the disadvantages of SEC are the co-isolation of protein aggregates with the same size range as EVs and time consumption. In addition, it is not suitable for batch processing because of the small sample volume loaded, which reduces the extraction efficiency of the exosomes [89]. Therefore, isolation of exosomes using SEC alone is not feasible, and SEC is usually combined with differential centrifugation or other methods [89,93]. Currently, SEC is mostly combined with ultracentrifugation for the extraction of milk exosomes [89,93]. In this case, larger EVs, milk fat globules, and other substances are first removed by ultracentrifugation; then, milk exosomes are separated by SEC [89]. In addition, SEC depends on gravity and low pressure to isolate milk exosomes, indicating that it can retain the unruptured regular shapes of milk exosomes [89,93].

#### 3.1.4. Polymer-Based Precipitation (with Polyethylene Glycol)

Polymer-based precipitation is a chemical precipitation method. Hydrophilic polymers such as polyethylene glycol combine with the water molecules surrounding exosomes such that particles with low solubility and exosomes can be precipitated by centrifugation [89,94,95]. Although time-consuming, precipitation using polyethylene glycol is easy and results in a more uniform size than that with ultracentrifugation [88]. However, disadvantages include low purity and the implementation of a post-clean-up process [88,93,95] because polyethylene glycol precipitates not only exosomes, but also other components such as nucleic acids, lipoproteins, and proteins [89,95]. Several commercial exosome isolation kits based on polymer precipitation methods have been developed. These include Total Exosome Isolation (Invitrogen, Carlsbad, CA, USA), ExoQuick Exosome Precipitation Solution (System Biosciences, Palo Alto, CA, USA), and EXO-prep (HansaBioMed, Tallinn, Estonia). The ExoQuick kit is one of the most commonly used kits for extracting exosomes from human serum, plasma, milk, and ascitic fluid. Zhou et al. extracted miRNAs from human breast milk using an ExoQuick kit. They showed that immune-related miRNAs were abundant in breast milk exosomes and were transferred from the mother to the infant [96]. However, this method is expensive and has a negative effect post-analysis because of the lipoproteins present in milk exosomes [6,89,93].

#### 3.1.5. Immunoaffinity

The immunoaffinity method for exosome isolation is based on the specific interaction between exosome membrane proteins and their corresponding antibodies [88,95]. At present, the immuno-magnetic beads technique is commonly used [88,89]. Antibody-coated beads specifically bind to exosomes, which distinguishes them from unbound impurities [88,89,95]. Exosome protein markers, such as CD9, CD63, and CD81, have been extensively used in commercial exosome isolation kits [6,95]. Sedykh et al. reported that extra-purified CD9^+^ and CD63^+^ exosomes were isolated from 18 horse milk samples using anti-CD9- and anti-CD63-Sepharoses [97]. The advantages of this method include a high specificity and the retention of intact structures. However, the limitations include high cost, low yield, harsh processes, difficult storage conditions, and unsuitability for processing many samples [89,93]. Therefore, it can be combined with ultracentrifugation or other methods to obtain better results [89,93].

#### 3.1.6. Microfluidic Devices

Microfluidic technology is a recently developed technique for high-throughput analyses [88,89]. Microfluidic chips allow for minimal sample volume and reagent consumption in their dedicated channels [88,89]. Microfluidic technology has many advantages for the separation of exosomes, such as short time, high efficiency, easy operation, and low cost [6,89,98]. Three types of microfluidic technology are in use and are categorized based on size, immunoaffinity, and dynamic separation [6,88,89]. Microfluidic chips based on size separation consist of nanofilters, membranes, and arrays [89]. After being trapped in these channels, exosomes are separated [89]. The microfluidic device with immunoaffinity has a microchannel with antibodies or affinity particles or immunomagnetic beads, resulting in milk exosomes being isolated by specific biomarkers in the microchannel [89]. Dynamic separation in microfluidic technology uses external forces such as electric, acoustic, and hydrodynamic forces [89]. Recently, new microfluidic-based exosome separation techniques have emerged [98].

### 3.2. Characterization Methods

Prior to further experiments, it is essential to confirm the morphology, size distribution, and concentration of milk exosomes in purified exosome solutions [87]. Several technologies have been used to characterize milk exosomes [88,93]. The most widely used technology is western blotting, which evaluates specific protein markers [88,93]. Nanoparticle tracking analysis technology detects the size and concentration of milk exosomes [88,93]. Nanoparticle tracking analysis tracks and analyzes particles using a built-in high-speed camera and software, resulting in particle size distribution and particle concentration analysis [93]. Electron microscopy techniques, such as transmission electron microscopy and scanning electron microscopy, are also used to examine size and morphology [88,93]. Additionally, atomic force microscopy and scanning probe microscopy can be used to estimate the size, structure, and topology of the surfaces of individual exosomes [88,93]. Dynamic light scattering is used to determine the size, surface charge, and exosome concentration [93]. In addition, flow cytometry and antibody-based exosomal arrays have been used to identify milk exosomes [93].

Most milk exosomes share common characteristics with other exosomes and have unique characteristics [93]. Milk exosomes have a diameter of 30–150 nm and have many surface markers on their membranes for identification [6,93]. The most enriched marker proteins are tetraspanins (CD9, CD63, and CD81), Alix, and flotillin1 [88,93]. According to proteomic profile analysis using Information-Dependent Acquisition Mass Spectrometry of human and bovine milk exosomes, four proteins (lactadherin, butyrophilin, perilipin-2, and xanthine dehydrogenase/oxidase) are commonly found [99]. Previously, bovine milk-derived EVs were reported to express exosome marker CD63, immunoregulatory miRNAs including miR-30A, -223, 92a, and milk-specific β-casein and β-lactoglobulin mRNA [100]. Along with the classic CD63, MFG-E8 has been reported as an exosome surface marker protein for milk exosomes [21,101]. Milk exosomes feature testilin, Rab-GTPase, Alix, and Tsg, which are involved in managing membrane fusion, interacting with cytoskeletal proteins, and endocytosis [97]. Most studies have reported that the solubility of milk exosomes depends on their pH [102]. Milk has a pH of 7.4; therefore, milk exosomes have a high solubility near this value [93]. Since the particle size of milk exosomes depends on pH, the particle size is the smallest at pH 5 and most uniform at pH 4.16 [93,103,104]. However, how pH affects the stability and size of milk exosomes remains unclear [88,93,98]. Furthermore, milk exosomes have superior biocompatibility as natural nanocarriers, owing to their liposomal- or lipid-based structures [103]. Milk exosomes can cross physiological barriers, including the blood–brain barrier [105]. Somiya et al. showed that milk exosomes extracted by acid treatment and ultracentrifugation were easily taken up by RAW264.7 macrophages [106].

## 4. Therapeutic Potential of Milk Exosomes Related to Musculoskeletal Health

### 4.1. Studies on Effects of Milk Exosomes on Bone and Muscle Health

Several recent studies (2017–2023) have investigated the beneficial effects of milk-derived exosomes on bone and muscle health (Table 1). All milk exosomes were obtained from bovine sources. In the human pre-osteoblastic MC3T3-E1, human osteoblast Saos-2, or osteoclastogenesis RAW264.7 cell lines, milk exosomes elicited osteoblast proliferation and differentiation as well as osteoclast differentiation inhibition [107,108,109,110]. Milk conditions are different for commercial pasteurized, colostrum, and fresh milk. Commercial pasteurized-milk-derived exosomes were used at the highest dose likely because pasteurization diminishes the exosome component compared to that in fresh milk. In in vivo tests, the treatment doses of pasteurized-milk and colostrum exosomes causing similar effects on bone mineral density in rat and mice models were significantly different; treatment doses of commercial pasteurized-milk exosomes were approximately 10-fold higher than that of colostrum exosome [107,109]. Three studies reported the muscle response to milk-derived exosomes [111,112,113]. We summarized the results by including one paper published in 2017 because there were few cell-level data such as C2C12 related to milk exosomes and muscle health. Using C2C12 myotubes, anabolic effects of milk exosomes on muscles were demonstrated by an increase in muscle protein synthesis and myotube diameter; however, the mTOR signaling pathways were not altered. Rather, the levels of bovine-specific miRNAs, including miR-149-3p and miR-2881, were upregulated by milk exosomes [111]. In 2017, Mobley et al. also reported that supplementing C2C12 myotube cell cultures with bovine milk exosomes promoted myotube growth, indicating a possible link between milk intake and muscle protein accretion [113]. In contrast, in in vivo studies using a young rodent model, feeding of exosomes and RNA-depleted diets for 6 weeks did not change grip strength in 3-week-old male and female C57BL/6 mice [112]. However, feeding of exosome- and RNA-depleted diets for 4 weeks increased muscle growth in the gastrocnemius muscles of 4-week-old male and female Fisher 344 rats (as determined by CSA), without changes in muscle weight or functional outcome (rotarod performance) [113]. A later study suggested that, despite a decrease in EV numbers, the enrichment of exosomal RNA levels in exosome- and RNA-depleted diets compared to those in the control diet may elicit anabolic effects in the muscle. In summary, compared to bones, where milk exosomes exerted a consistently significant effect on bone growth (i.e., osteoblast proliferation and differentiation) in cell and animal models, the muscle health effect was not consistent. Therefore, there are not many studies published yet on the beneficial effects of milk exosomes on muscles, so the review is limited, and it is necessary to monitor the results of continued research in the future.

### 4.2. Studies on Anti-Inflammatory and Antioxidant Action of Milk Exosomes

As inflammation and oxidative stress are the underlying mechanisms involved in musculoskeletal disorders, studies reporting the antioxidant and anti-inflammatory properties of milk exosomes have been summarized (Table 2 and Table 3). In the literature published between 2017 and 2023, milk exosomes were isolated and characterized by ultracentrifugation in most papers, except for He et al., who performed isolation and characterization using a total exosome isolation kit [114]. In the reviewed studies, milk exosome treatment consistently lowered TNF-α levels. In contrast, canine colostrum milk treatments showed a significantly increased production of IL-12p40, IL-6, IL-8, MCP-1, and stem cell factor in mesenchymal stem cells derived from bone marrow (cBM-MSCs) and IFN-γ, IL-8, MCP-1, TNF-α, and β-nerve growth factor in mesenchymal stem cells derived from adipose tissue (cAd-MSCs) [115]. In the first study using canine colostrum milk, the treatments of exosomes derived from canine colostrum milk increased the secretion of IL-8 and MCP-1, which are factors related to migration, chemotaxis, and angiogenesis in both cBM-MSCs and cAd-MSCs [115]. In addition, canine colostrum milk exosomes had also a potent antioxidant effect by decreasing reactive oxygen species (ROS) activity.

TNF-α is known as a pleiotropic cytokine with important but sometimes contradictory functions in numerous physiological processes related to immunity and inflammation [117,118]. In addition, IL-6 is a pleiotropic cytokine with a key role in various biological processes such as regulation of the immune response, inflammation, haematopoiesis, apoptosis, cell survival, and cell proliferation [117,118]. Treatment with milk exosomes from various sources showed a common tendency to reduce TNF-α and IL-6 levels in the investigated papers. In Mdr1a-/- mouse models, milk exosome- and RNA-sufficient diets lowered the serum concentration of C-X-C motif ligand 9 compared to that by milk exosome- and RNA-depleted diets. Moreover, milk exosome- and RNA-depleted diets lowered the concentration of miR-200a-3p in the liver and ceca [84]. These results imply that milk exosomes and miRNA depletion exacerbate cecal inflammation. Bovine milk, especially colostrum-derived exosomes in a murine model of ulcerative colitis induced by dextran sodium sulfate, activated the proliferation of colonic epithelial cells and macrophages and created an environment to relieve inflammation by effectively removing ROS and regulating the expression of immune cytokines [119]. ROS generation is a representative factor that can be used to investigate oxidative stress; the milk exosomes investigated in this review consistently reduced ROS levels. Human-breast-milk-derived exosomes exert antioxidant activity as shown by the increased viability of intestinal epithelial cells treated with H_2_O_2_ [55,124]. In addition, Wang et al. assessed the protective effects of bovine milk exosomes against oxidative stress in intestinal crypt epithelial cells (IEC-6), subsequent to H_2_O_2_ treatment. They reported increased levels of superoxide dismutase and glutathione peroxidase and decreased levels of ROS, lactate dehydrogenase, and malondialdehyde (MDA) [121]. Bovine milk exosomes increased intracellular miR-146a and miR-155 levels and inhibited the expression levels of nuclear factor erythroid 2-related factor 2 (Nrf2) and heme oxygenase 1 (HO-1) genes, but promoted HO-1 protein expression [121]. Consistent with the results of other studies, the MDA levels were increased by camel milk exosomes in male albino rats [71]. The mechanisms underlying these oxidative stresses involve Wnt signaling, which has been proposed as the target pathway upregulated by milk exosomes [124].

## 5. Conclusions

Milk exosomes, which are dietary exosomes, have been studied for drug delivery, particularly in the development of anti-cancer drugs. Recently, as the average life expectancy has increased, studies on musculoskeletal diseases related to the muscles and bones, such as sarcopenia and osteoporosis, are increasing. Herein, we investigated the definition, source, cargo, isolation, and characterization of milk exosomes, literature on milk-exosome studies on musculoskeletal diseases, and antioxidant and anti-inflammatory properties of milk exosomes, since inflammation and oxidative stress are underlying mechanisms involved in musculoskeletal disorders. Research on the molecular mechanisms of milk exosomes and musculoskeletal diseases will be helpful for the development of delivery mechanisms and disease treatments. Therefore, the potential protective effects of milk exosomes against musculoskeletal diseases, inflammation, and oxidant stress are also expected to improve milk consumption as a source of milk exosomes.

## Figures and Tables

**Table 1:** Summary of reports on the beneficial effects of milk exosomes on bone and muscle health.

Source of Milk Exosome	Isolation/Characterization	Model	Dose of Milk Exosome	Duration of Milk Exosome Treatments	Major Outcomes (by Exosome Treatment) ^1^	Application ^1^	Ref.
**Bone**							
Bovine commercial pasteurized milk	Ultracentrifugation/-	Human osteoblast Saos-2 cells Human preosteoblastic MC3T3-E1 cells	1, 10, 100, 200, 500, 1000 μg/mL	24, 48, 72 h	↑Runx2, Osterix (key transcription factor for osteoblast differentiation)	↑Osteoblast differentiation	[109]
		Female Sprague-Dawley rats (6-week-old)	50 mg/kg/day	2 weeks	↑Trabecular and cortical volumetric bone mineral density of the tibia	↑Bone mineral density	
Bovine colostrum	Ultracentrifugation/-	Human preosteoblastic MC3T3-E1 cells	0.1 μg/mL	12 h	↑ALP, Runx2 ↑STAT5a, GJA1	↑Osteoblasts proliferation and differentiation	[108]
Bovine fresh milk	Ultracentrifugation/TEM, NTA	Human preosteoblastic MC3T3-E1 cells	20 μg/mL	72 h (treated every 24 h)	↑ALP and OPN ↑CCK8 ↑GJA1	↑Osteoblasts proliferation and differentiation	[110]
		Male C67BL/6 mice (8-week-old) (mice skull operation model)	1.2 μg plus 100 μL GelMA	2 weeks	↑BMP-2	↑Bone repair promotion	
Bovine colostrum	Ultracentrifugation/SEM, TEM	Raw 264.7 cells (RANKL- plus M-CSF-induced Osteoclastgenesis cell model) Human preosteoblastic MC3T3-E1 cells	50, 150 ng/mL 20, 50, 100, 150, 300, 500 ng/mL	-	↓ Number of osteoclast (TARP-positive) = Cytotoxicity	↓Osteoclast differentiation -	[107]
		Male C67BL/6 mice (24-week-old) (Glucocorticoid-induced osteoporosis model)	0.15, 1.5 mg/kg/day	8 weeks (before osteoporosis induction)	↑ Femur bone mineral density, percent bone volume	↑Osteoporosis prevention ↑Bone remodeling improvement ↑Inhibition of bone resorption	
**Muscle**							
Bovine milk (whey protein)	Ultracentrifugation/TEM	C2C12 cells	1, 5, 10 mg/mL	24 h	↑ Myotube diameter ↑miR-149-3p, miR-2881, miR-214, miR-30b, miR-16b ↑p-AMPKα = mTOR, p-p70s6k1, rpS6 ↓MyoD	↑Muscle anabolism (protein synthesis)	[111]
Bovine colostrum	Ultracentrifugation/-	Female/male C57BL/6 mice (3-week-old)	ERD diet including 0.5 L milk ERS diet including 0.5 L milk	6 weeks	= Grip strength, amino acid profiles in muscle = Calcium, markers of liver and kidney ↑Tmem100 (in female ERD group) ↓Rhobtb1 mRNA and Socs2 mRNA (in ERD group)	-	[112]
Bovine colostrum	EVs isolation kit/NTA	Female/male Fisher 344 rats (5-week-old including adaptation period 1 week)	ERD diet ERS diet	4 weeks	↓Total food consumed ↑Gastrocnemius mitochondrial ROS emission ↑Gastrocnemius fCSA (in ERD group) ↑miRNA 2887-1 and miRNA 885	↓Gastrocnemius muscle anabolism	[113]

^1^ ↑, increase; ↓, decrease; =, no change; -, not determined. Abbreviations: Runx2, Runt-related transcription factor 2; ALP, alkaline phosphatase; STAT, signal transducer and activator of transcription; OPN, osteopontin; GJA1, gap junction protein alpha 1; BMP, bone morphogenetic protein; AMPK, phosphorylated adenosine monophosphate-activated protein kinase; mTOR, mammalian target of rapamycin; MyoD, myogenic differentiation protein; TEM, transmission electron microscopy; SEM, scanning electron microscopy; EVs, extracellular vesicles; NTA, nanoparticle tracking analysis; M-CSF, macrophage-colony-stimulating factor; ERD, an exosome and RNA-depleted; ERS, an exosome and RNA-sufficient; miRNA, microRNA; fCSA, fiber cross sectional area.

**Table 2:** Summary of reports on the beneficial anti-inflammatory effects of milk exosomes.

Model	Source of Milk Exosome	Isolation	Dose of Milk Exosome	Duration of Milk Exosome Treatments	Major Outcome (by Exosome Treatment) ^1^	Ref.
Canine mesenchymal stem cells (derived from bone marrow and adipose tissue)	Canine colostrum	Ultracentrifugation	305.60 ± 46.7 μg/mL	-	↑IL-12p40, IL-6, IL-8, MCP-1, SCF (in bone marrow) ↑IFN-γ, IL-8, MCP-1, TNF-α, NGF-β (in adipose tissue)	[115]
NCM 460 cells (treated by LPS, IFN-γ)	Bovine colostrum	Ultracentrifugation	0.1 mg/mL	24 h	↓TNF-α, IL-10, IL-6, iNOS	[116]
RAW 264.7 cells (treated by LPS, IFN-γ)	Bovine colostrum	Ultracentrifugation	0.1 mg/mL	24 h	↓TNF-α, IL-10, IL-6, iNOS	
Ulcerative colitis (treated by DSS, 8 weeks balb/c mice)	Bovine colostrum	Ultracentrifugation	50 mg/kg	27 days	↓DSS-induced colitis, intestinal inflammation (ROS, iNOS, TNF-α, IL-6) ↑IL-10	
Ulcerative colitis (Intestinal-specific kindlin 2 knockout mice, C57BL/6 male)	Commercial cow milk	Ultracentrifugation	33 μg/g	4 days	↓Stool score, colon weight with fecal content, colon weight score, colon length score ↑Colon length	[114]
Caco-2 cells (treated by IFN-γ, LPS)	Cow milk	Ultracentrifugation	-	24 h	↓CXCL8, IL-1β, TNF-α, IL-12A, IL-23A, TGF-β1, NOS2, MMP9	[117]
THP-1 cells (treated by IFN-γ, LPS)	Cow milk	Ultracentrifugation	-	24 h	↓IL-6, IL-8, IL-12	
Human dental pulp stem cells (treated by LPS)	Human milk	Ultracentrifugation	200 μg/mL	24 h	↓TNF-α, IL-1β, IL-6	[118]
Male albino rats (treated by cyclophosphamide)	Camel milk	Ultracentrifugation	1.25 mg/kg	2 weeks	↓IFN-Y, CD4+, CD8+, IL-6, TNF-α	[41]
MCF7 cell (treated by cyclophosphamide)	Camel milk	Ultracentrifugation	20 mg/kg 1.25 mg/kg	4 weeks	↓IL-1β, NF-κB, inflammation	[119]
Necrotizing enterocolitis (5–9 days C57BL/6 mice)	Bovine milk	Ultracentrifugation	1 ng/mL	-	↓NLRP3 inflammasome, NF-κB signaling, TLR4, NLRP3, Casp1, IL-1β, H3	[81]
Treated by NEC (Necrotizing enterocolitis induced, 5 days C57BL/6 mice)	Human milk	Total Exosome Isolation kit	-	-	↓TNF-α, IL-1β ↑Epithelia tight-junction protein	[58]
Treated by hypoxia, garage feeding, LPS (C24B/6 mouse)	Human milk	Ultracentrifugation	-	-	↓Intestinal damage, IL-6, inflammation ↑Mucous production	[56]
H1299 cell (treated by LPS)	Bovine colostrum (curcumin-loaded)	Ultracentrifugation	25 μM	4 h	↓TNF-α, NF-kB activity	[11]
Treated by Caski cell (5~6 weeks female Athymic nude mice)	Bovine colostrum (curcumin-loaded)	Ultracentrifugation	20 mg/kg	7 weeks	↓TNF-α	
H1299 and MCF7 (treated by TNF-α)	Cow milk (Anthos-loaded)	Ultracentrifugation	8 mg/kg	8 weeks	↓TNF-α, NF-kB activity	[120]
RAW264.7 macrophages (LPS-induced)	Cow milk (astaxanthin-loaded)	Ultracentrifugation		24 h	↓IL-1β, IL-6, IL-12, TNF-α	[105]
HepaRG	Camel milk (colostrum, early, mid, late lactation periods)	Ultracentrifugation	20.62 ± 1.02, 29.29 ± 1.30, 35.94 ± 1.50, 36.87 ± 1.45 µg/mL, respectively	24 h	↓TNF-α, NF-kB, TGFβ1, COX-2	[40]
Dextran Sulfate Sodium-induced colitis (8 weeks balb/c mice)	Cow milk Human milk	Ultracentrifugation	50 mg/kg	6 days	↓Inflammation score, lymphocyte infiltration ↓TNF-α, IL-6 ↑TGF-β1	[121]

^1^ ↑, increase; ↓, decrease; =, no change; -, not determined. Abbreviations: IL, Interleukin; MCP-1, monocyte chemoattractant protein-1; TNF-α, tumor necrosis factor-alpha.; NGF-β, beta-nerve growth factor; SCF, stem cell factor; IFN-γ, interferon-gamma; iNOS, inducible nitric oxide synthase; ROS, reactive oxygen species; LPS, lipopolysaccharide; NF-κB, nuclear factor kappa B; MMP, matrix metalloproteinase; TLR, Toll-like receptor; NLRP, nod-like receptor family pyrin domain containing; Casp, caspase; TGF, transforming growth factor; COX-2, cyclooxygenase-2.

**Table 3:** Summary of reports on the beneficial effects of milk exosomes against antioxidant stress.

Model	Source of Milk Exosome	Isolation	Dose of Milk Exosome	Duration of Milk Exosome Treatments	Major Outcome (by Exosome Treatment) ^1^	Ref.
Canine mesenchymal stem cells	Canine colostrum	Ultracentrifugation	305.60 ± 46.7 μg/mL	-	↓ROS	[115]
HepG2 cells	Camel milk	Ultracentrifugation	6.17, 12.34, 24.68 µg/mL	24 h	↑NrF2, HO-1	[122]
CaCo2 cells	Camel milk	Ultracentrifugation	3.60, 7.20, 14.40 µg/mL	24 h	↑NrF2, HO-1	
Intestinal crypt epithelial cells (treated by H_2_O_2_)	Bovine milk	Ultracentrifugation	400, 800 μg/mL	24 h, 48 h	↑SOD, GPx, miRNA-146a, miRNA-155, HO-1 protein, Nrf2 ↓LDH, ROS, MDA	[123]
IEC-6 cells (treated by H_2_O_2_)	Human breast milk	Ultracentrifugation	0.1–10 μg/mL	-	↑Cell viability in oxidative stress	[55]
Intestinal stem cells (treated by H_2_O_2_)	Human milk	Ultracentrifugation	0.5 mg/mL	-	↑Axin2, c-MYC, Cyclin D1, Hes1, Dll1, Dll4	[124]
Caco-2 cells (treated by LPS, NCM460)	Human milk	Total Exosome Isolation kit	-	-	↓ZO-1, claudin-1, occludin	[58]
Male albino rats (treated by cyclophosphamide)	Camel milk	Ultracentrifugation	1.25 mg/kg 2.5 mg/kg	2 weeks	↑CAT, SOD, GPx ↓MDA	[41]
Rat spleen (treated by cyclophosphamide)	Camel milk	Ultracentrifugation	1.25 mg/kg	2 times (the first on day 5 and the second on day 10)	↑CAT, SOD, GPx ↓MDA	
Diabetic nephropathy (treated by streptozotocin, male albino rat)	Camel milk	Ultracentrifugation	10 mL/rat/day	12 weeks	↓MDA ↑CAT, SOD, GPx ↑TGFβ1, ICAM1, ETS1, ITGβ2, TIMP2, KIM1	[125]
Rat renal tissue (treated streptozotocin)	Camel milk	Ultracentrifugation	1.25 mg/kg BW	1 time per week for 1 month	↓MDA, ROS ↑CAT, SOD, GPx	
Female albino rats’ tumor tissue (injected by MCF7 cells)	Camel milk	Ultracentrifugation	20 mg/kg (orally)	4 weeks	↓MDA, iNOS ↑CAT, SOD, GPx	[119]
EVs and RNA-depleted fisher 344 rats	Bovine colostrum	--	-	4 weeks	↓ROS ↑GPx1	[113]
C24B/6 mouse (treated by hypoxia, garage feeding, LPS)	Human milk	Ultracentrifugation	-	-	↓Intestinal damage, MPO activity	[56]
HUVECs cells (treated methylglyoxal)	Milk	Ultracentrifugation	-	-	↓ROS ↑Nrf2, HO-1	[126]
RAW264.7 cells, NCM460 (treated by H_2_O_2_)	Bovine colostrum	Ultracentrifugation	0.1 mg/mL	24 h	↓ROS	[116]
In vitro free radical scavenging assay	Low-temperature pasteurized fat free milk	Ultracentrifugation	10^8^, 10^9^, 10^10^ particles	15 min	↑ABTS radical scavenging activity	[85]

^1^ ↑, increase; ↓, decrease; =, no change; -, not determined. Abbreviations: ROS, reactive oxygen species; Nrf2, nuclear factor erythroid-2-related factor 2; HO-1, heme oxygenase-1; SOD, superoxide dismutase; GPx, glutathione peroxidase; miRNA, microRNA; LDH, lactate dehydrogenase; MDA, malondialdehyde; ZO-1, zonula occludens-1; CAT, catalase; TGF, transforming growth factor; ICAM, intercellular adhesion molecules; ETS1, transformation specific 1; ITGβ2, integrin subunit beta 2; TIMP, tissue inhibitors of matrix metalloproteinase; MPO, myeloperoxidase; ABTS, 2,2′-azino-bis(3-ethylbenzothiazoline-6-sulfonic acid; Hes1, Hes Family BHLH Transcription Factor 1; Dll1, Delta Like Canonical Notch Ligand 1; Dll4, DLL4 delta like canonical Notch ligand 4.

## Data Availability

Not applicable.
